# Ab Initio Study of Formation Mechanisms and Thermochemical Properties of Reactive Oxygen Species (ROS) in Photocatalytic Processes

**DOI:** 10.3390/ijms26188989

**Published:** 2025-09-15

**Authors:** Silvia González, Ximena Jaramillo-Fierro

**Affiliations:** Departamento de Química, Facultad de Ciencias Exactas y Naturales, Universidad Técnica Particular de Loja, San Cayetano Alto, Loja 1101608, Ecuador; sgonzalez@utpl.edu.ec

**Keywords:** reactive oxygen species, photocatalysis, quantum chemical calculations, MP2 method, thermochemical properties

## Abstract

This study explores the thermochemical properties and formation mechanisms of reactive oxygen species (ROS) relevant to photocatalytic processes, aiming to clarify their molecular characteristics and reaction dynamics. The research focuses on key ROS, including the superoxide anion radical (^•^O_2_^−^), hydrogen peroxide (H_2_O_2_), singlet oxygen (^1^O_2_), and hydroxyl radical (^•^OH), employing Møller–Plesset second-order perturbation theory (MP2)-level quantum chemical calculations. Solvent effects were modeled using water to simulate conditions commonly found in photocatalytic environments. The computed energetic profiles and stabilities of the ROS offer insights into their relative reactivities and possible interconversion pathways. These findings enhance the understanding of how ROS behave under photocatalytic conditions, with implications for their role in degradation mechanisms and redox cycles. Overall, the results support the development and optimization of photocatalytic technologies for environmental applications, including pollutant degradation and disinfection of water and air.

## 1. Introduction

Photocatalysis utilizes light energy to drive chemical reactions by generating electron/hole pairs (e−/h+) within a semiconductor catalyst [1]. These photogenerated charge carriers interact with oxygen and water molecules, leading to the formation of reactive oxygen species (ROS) such as superoxide anion radical (^•^O_2_^−^), hydrogen peroxide (H_2_O_2_), singlet oxygen (^1^O_2_), and hydroxyl radical (^•^OH) [2,3,4]. These species exhibit distinct redox properties, enabling their participation in oxidation-reduction reactions relevant to environmental and biological processes [5,6,7,8,9,10]. However, their reactivity and stability strongly depend on external factors, including oxygen concentration, pH and the presence of catalytic sites [11,12]. Recent studies have shown that structural modifications of photocatalysts, such as incorporating metal-semiconductor heterojunctions, can optimize ROS production and regulation, favoring specific reaction pathways and improving efficiency in contaminant degradation [13,14].

ROS formation in photocatalysis primarily occurs through electron transfer reactions and energy transfer mechanisms [15]. Superoxide anion (^•^O_2_^−^) is typically formed by the single-electron reduction in molecular oxygen (O_2_) facilitated by conduction band electrons from the photocatalyst [16]. In contrast, singlet oxygen (^1^O_2_) arises from an energy transfer process, where an excited photocatalyst transfers energy to molecular oxygen, altering Its spin state to form a highly reactive species [17]. Hydrogen peroxide (H_2_O_2_) can be generated either by the interaction of the superoxide radical (^•^O_2_^−^) with protons and electrons or through the recombination of hydroxyl radical (^•^OH). Among these species, hydroxyl radical (^•^OH) is the most reactive, capable of oxidizing almost any organic or inorganic molecule. Its formation is often associated with water oxidation by valence band holes or via interactions with pre-existing ROS, such as superoxide anions and hydrogen peroxide [18,19]. In heterogeneous photocatalysis, pH-dependent speciation and redox potentials govern ROS interconversion (e.g., HO_2_^•^/O_2_•^−^ equilibrium; Nernstian shifts), and interfacial adsorption/charge can dominate the apparent reactivity (e.g., ^•^OH adsorbed as trapped holes; peroxo-like bridged surface species/H_2_O_2_) [20]. These experimentally established features provide the context in which we interpret the thermodynamic maps presented in this study.

The reactivity of ROS makes them critical in natural and engineered systems. In the environment, ROS are key players in biogeochemical cycles, affecting pollutant degradation and redox equilibria in water and atmospheric systems. Recent studies highlight how oxygen concentration and transition metals influence ROS formation, particularly in aquatic and atmospheric environments [21].

The generation and transformation of ROS in the environment play a fundamental role in biogeochemical cycles and the natural degradation of contaminants [22]. Recent studies have highlighted the influence of environmental factors such as oxygen concentration and the presence of transition metals on ROS formation in aquatic and atmospheric media [23]. Additionally, the development of high-entropy oxides has been identified as a promising strategy for the efficient activation of molecular oxygen in sustainable photocatalytic processes, promoting selective ROS generation and reducing the need for expensive catalysts [24]. Beyond their application in decontamination processes, ROS have found utility in biomedical fields, particularly in therapies based on the selective cytotoxicity of these species. Nanoplatforms that generate ROS have been reported for cancer treatment, where these species induce apoptosis through controlled oxidative stress mechanisms [25].

Reactive oxygen species (ROS) are important intermediates in oxidation and reduction reactions that occur near the surface of photocatalysts such as titanium dioxide (TiO_2_) [26,27,28], zinc titanate (ZnTiO_3_) [29], zinc oxide (ZnO) [30], and others. These processes include the oxidation of organics and reduction in inorganic molecules [31,32]. Thus, efficient generation of ROS is fundamental for optimizing the efficiency of photocatalytic processes [33,34]. The adsorption of molecules on the catalyst surface is not a prerequisite for efficient oxidation-reduction. In fact, the oxidation and reduction reactions that occur by photooxidation could occur in the bulk solution where ROS plays a key role. In this way, the contribution of ROS located on the surface of the catalyst is reduced [35]. Literature suggests that photocatalytic generation of ROS on TiO_2_ surfaces predominantly occurs through reactions at the anionic bridge OH site and the cationic terminal OH site. At the anionic bridge OH site, a proposed mechanism involves nucleophilic oxidation of water, where a photoinduced hole targets the O(^2−^) bridge, generating a hydroxyl radical (^•^OH). Conversely, at the cationic terminal OH site, where positively charged holes are unreactive, it is proposed that a photoinduced electron trapped at the TiO_2_ surface reduces O_2_, forming the superoxide anion radical (^•^O_2_^−^) [36].

Despite extensive interest in surface ROS reactions due to their diverse chemical applications, there remains a notable lack of detailed understanding regarding the thermochemical properties and generation pathways of several ROS, including the superoxide anion radical (^•^O_2_^−^), hydrogen peroxide (H_2_O_2_), singlet oxygen (^1^O_2_), and hydroxyl radical (^•^OH). The stability and generation pathways of reactive oxygen species (ROS) are influenced by their thermochemical properties, which can be difficult to determine by experimental methods due to their short half-lives and their tendency to rapidly transform into other species because of their high reactivity. In this way, computational methods are a valuable tool to evaluate the stability and formation mechanisms of these species, as well as their possible transformations in various processes [35].

Methods such as Density Functional Theory (DFT) and Møller–Plesset second-order perturbation theory (MP2) have been widely used to evaluate the stability and reactivity of those species and systems. Recent studies have proposed improvements in MP2 calculations for the prediction of weak interactions, providing a better approximation of experimentally observed phenomena [37]. Additionally, advanced techniques such as Laplace-transformed MP2 have optimized the simulation of periodic systems, allowing for more accurate assessments of photocatalytic materials [38]. MP2 calculations can elucidate the stability of ROS intermediates by determining their energetic profiles and relative stabilities compared to those of other species. Therefore, this MP2 study investigated the thermochemical properties and formation reactions of key ROS generated during photocatalytic processes: the superoxide anion radical (^•^O_2_^−^), hydrogen peroxide (H_2_O_2_), singlet oxygen (^1^O_2_), and hydroxyl radical (^•^OH). Understanding these properties and reactions can disclose potential pathways for ROS generation and control their reactivity.

Recent literature reviews highlight the importance of ROS generation and regulation in photocatalysis, not only for contaminant degradation but also for emerging applications in biomedicine and energy conversion. This study fits within this context, addressing the stability and reactivity of ROS through advanced computational methodologies and exploring their applicability in environmental remediation processes and sustainable energy production. The combination of experimental and theoretical strategies will help advance the understanding of these processes and the development of more efficient and sustainable technologies [22,25,37,38].

## 2. Results

### 2.1. O*_2_* Species

Table 1 summarizes the thermochemical and molecular properties of O_2_(T), ^1^O_2_(S), and ^•^O_2_^−^(D). The units for Gibbs free energy (G^°^) and total energy (E^°^) are expressed in atomic units of energy (a.u.e) o hartrees (ha). Bond distances (d_O-O_) are in Angstroms (Å); the dipole moment (μ), indicating directional charge distribution polarizations, is in Debyes; electronic spatial extent (R^2^) in Angstroms squared (Å^2^), and the vibrational frequency (ν_O-O_) is in inverse centimeters (cm^−1^).

#### Formation Reactions of O_2_ Species

The photogeneration of radical oxygen species, specifically singlet oxygen ^1^O_2_(S) and the superoxide radical ^•^O_2_^−^(D) from triplet oxygen O_2_(T), the most abundant and stable form of oxygen, can follow two distinct pathways. The calculated reaction energy values for these pathways are presented in Table 2. Reaction (3) is the inverse of Reaction (2). Both are included because they illustrate the obtention of two different species, ^•^O_2_^−^(D) and ^1^O_2_(S), each of which participates in other reactions to obtain other ROS.

**Figure 1:**
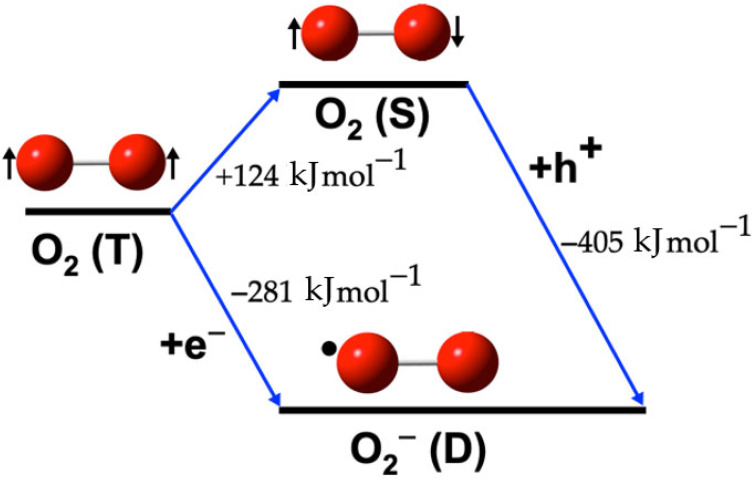
Calculated pathways for the formation of ^•^O_2_^−^(D) species. The blue arrows indicate the transformations between species.

Figure 1 illustrates the calculated pathways for the formation of the ^•^O_2_^−^(D) species. In this figure, blue arrows indicate the transformations between species. Singlet oxygen ^1^O_2_(S) is represented with a pair of electrons in opposite directions, triplet oxygen O_2_(T) with two unpaired electrons in the same direction, and doublet oxygen ^•^O_2_^−^(D) is depicted with a black sphere, indicating its radical nature.

Several reactions involving ROS are initiated by the superoxide radical anion ^•^O_2_^−^(D). This species exhibits high oxidative activity, although selective and medium-dependent. The combination of its redox couples and pH-dependent speciation (HO_2_^•^/^•^O_2_^−^ equilibrium) accounts for its reactivity toward suitable substrates; in particular, protonation (pK_a_ ≈ 4.8) and HO_2_^•^/^•^O_2_^−^ disproportionation supports both the stronger oxidizing character at acidic pH and the marked pH dependence [39]. This behavior has been verified experimentally: (i) in the aerobic degradation of p-nitrophenol, EPR spin-trapping and quenching assays identify ^•^O_2_^−^ as the key species with preferential attack at the nitro group, and (ii) in heterogeneous photocatalysis, measurable kinetics with phenols show Hammett-type substituent correlations for ^•^O_2_^−^-mediated oxidation, evidencing effective yet selective reactivity [20,39,40]. Moreover, its longer lifetime in water relative to ^•^OH favors participation in multiple downstream pathways, including H_2_O_2_ formation via disproportionation, reinforcing its role as an active and versatile oxidant in ROS networks under realistic photocatalytic conditions [39]. Table 3 presents the calculated energy values for each reaction involving the ^•^O_2_^−^(D) species, along with the resulting products.

As indicated in Table 3, Reaction (5) exhibits negative ΔE and ΔG values, confirming a thermodynamically favorable process, whereas Reaction (6) shows positive values, indicating that its direct occurrence is not favored. In photocatalytic systems, the formation of ^•^O_2_H is therefore expected to proceed via coupled proton/electron-transfer pathways rather than a single elementary step, in agreement with previous studies [41].

### 2.2. OH Species

Table 4 presents a summary of the thermochemical and molecular properties of the hydroxyl ion OH^−^(S) and the hydroxyl radical ^•^OH(D). Between parenthesis, the multiplicity of each species is described (S = singlet and D = doublet). The (^•^) symbol indicates that the species is radical and the negative sign as super-index indicates that the species has negative charge.

#### Formation Reactions of OH Species

**Figure 2:**
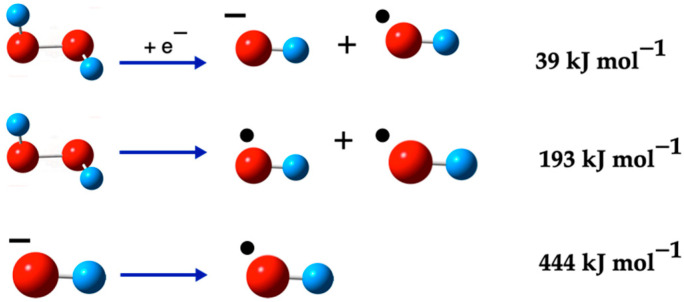
^•^OH formation from OH^−^ ion and H_2_O_2_. The red balls are oxygen atoms and small blue balls are hydrogen atoms, the small black ball mark OH as a radical, and the negative sign as super-index indicates that the species has negative charge.

The formation of the hydroxyl radical (^•^OH) from hydrogen peroxide (H_2_O_2_) involves varying energy requirements depending on the reaction pathway. Figure 2 illustrates the various routes for obtaining both the ^•^OH and the OH^−^ ion from H_2_O_2_. This figure provides a visual representation of the different energy pathways and their respective products, offering a clearer understanding of the reaction mechanisms involved in the formation of these important chemical species.

### 2.3. H*_2_*O*_2_* Species

H_2_O_2_ is known as a mild oxidizing agent, capable of oxidizing a variety of organic and inorganic compounds. Interestingly, it is the only stable reactive oxygen species (ROS) and can be specifically detected following the decomposition of other ROS [42,43]. Table 5 details the molecular properties of these three forms of H_2_O_2_. Notably, the fourth column highlights that the formation of H_2_O_2_^−^ is spontaneous, whereas H_2_O_2_^+^ formation is non-spontaneous. The structural differences and vibrational frequency values, as shown in this table, stem from their conformational differences.

**Figure 3:**
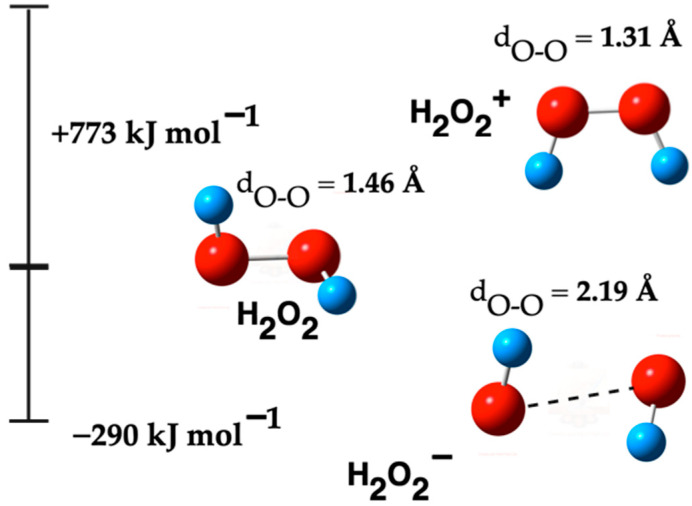
ΔG^°^ of H_2_O_2_ species. The red balls are oxygen atoms and small blue balls are hydrogen atoms. The dot line in H_2_O_2_^−^ species indicates that both oxygen atoms are unbonded.

Figure 3 illustrates the optimized molecular structures for the H_2_O_2_ species and shows the Gibbs free energy differences between neutral H_2_O_2_ and the respective ionic forms (H_2_O_2_^−^ and H_2_O_2_^+^). The computed total spin populations are 1.75 for H_2_O_2_^+^ and 0.50 for H_2_O_2_^−^.

#### 2.3.1. Formation Reactions of H_2_O_2_ Species

Hydrogen peroxide (H_2_O_2_) is typically generated in photocatalysis as a byproduct of water (H_2_O) oxidation in the presence of active ROS like hydroxyl radicals (^•^OH) and superoxide anions (^•^O_2_^−^) [44]. In contrast, the Fenton reaction, involving the reduction of H_2_O_2_ by iron ions (Fe^2+^) under light, is a pathway for the consumption of preformed H_2_O_2_ [45,46,47]. Table 6 summarizes several reactions for H_2_O_2_ generation.

The results of this study suggest that Reactions (12) and (16) allow the exergonic formation of H_2_O_2_ from the superoxide radical (^•^O_2_^−^) radical.

**Figure 4:**
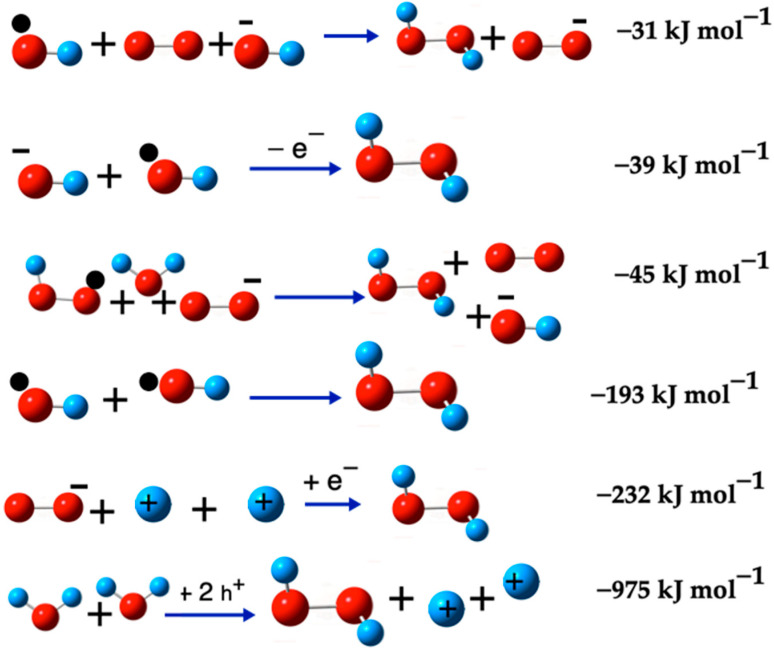
Some H_2_O_2_ formation reactions. The red balls are oxygen atoms, and the small blue balls are hydrogen atoms, the small black ball marks ^•^O_2_^−^(D), ^•^OH and ^•^O_2_H as radicals. The O_2_(S) is marked with two small black arrows and H+ cation is marked with a + sign.

Figure 4 shows the H_2_O_2_ generation reactions that are presented in Table 6 as thermodynamically favorable (ΔG < 0).

**Figure 5:**
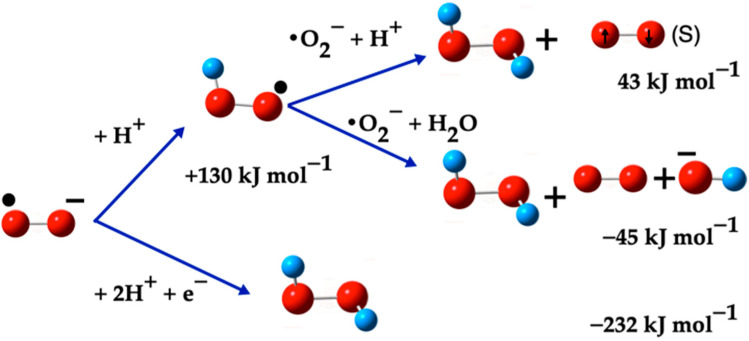
H_2_O_2_ formation from the ^•^O_2_^−^(D). The red balls are oxygen atoms and small blue balls are hydrogen atoms, the small black ball marks ^•^O_2_^−^(D) and ^•^O_2_H as radicals. The O_2_(S) is marked with two small arrows and the (S) symbol.

Figure 5 shows two pathways originating from the superoxide radical ^•^O_2_^−^ and require an acidic medium for them to take place. On the one hand, in the presence of an H^+^ ion, which can form spontaneously in an aqueous medium, the ^•^O_2_^−^ radical reacts with the H^+^ ion to form the intermediate species, ^•^O_2_H (−45 kJ mol^−1^). This species then reacts with an H^+^ ion and with the superoxide ^•^O_2_^−^ present in the medium, to form O_2_(S) and H_2_O_2_ (43 kJ mol^−1^). On the other hand, the ^•^O_2_^−^ radical, two H^+^ ions, and one electron led to the spontaneous formation of H_2_O_2_ (−232 kJ mol^−1^).

Hence, both pathways for the formation of H_2_O_2_ require an acidic medium. The presence of a single electron contributes to the spontaneity of these reactions. The main pathways for generating H_2_O_2_ from the superoxide radical (^•^O_2_^−^) are illustrated in Figure 5, specifically through Reactions (10), (12), and (16).

#### 2.3.2. Dissociation Reactions of H_2_O_2_ Species

The complexity of ROS studies lies in the numerous possible species and their interactions with electron/hole (e^−^/h^+^) pairs, leading to the formation, recombination, and dissociation of various ROS due to their high chemical activity. Table 7 outlines some reactions for generating various ROS from H_2_O_2_ dissociation.

**Figure 6:**
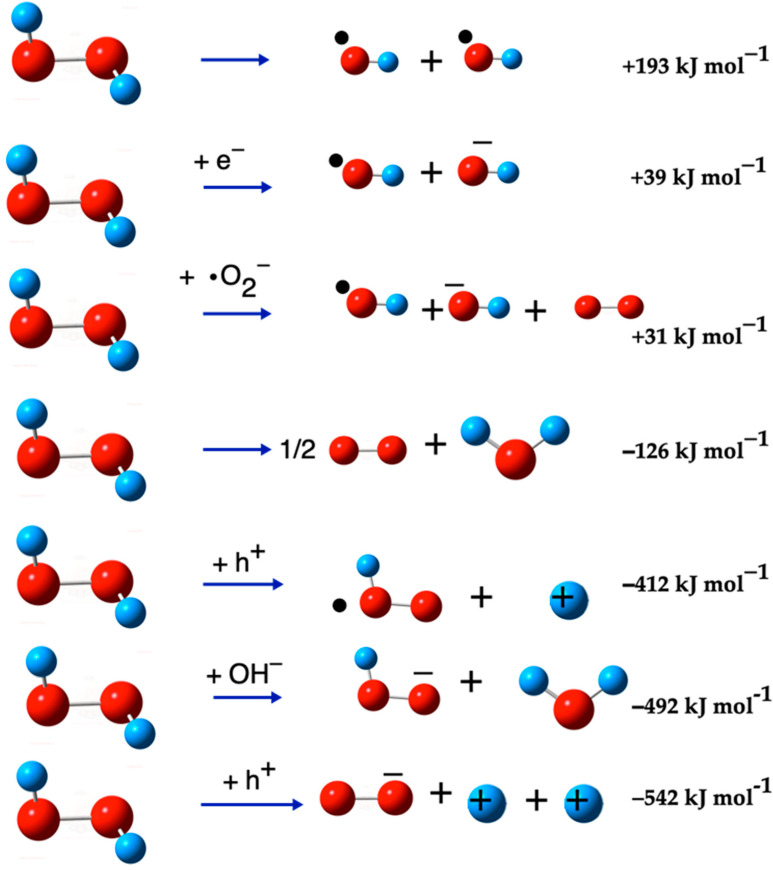
ROS generation from H_2_O_2_ dissociation. The red balls are oxygen atoms, and the small blue balls are hydrogen atoms, the small black ball marks OH as a radical, and the negative sign as super-index indicates that the species has negative charge.

Figure 6 visually represents the H_2_O_2_ dissociation reactions listed in Table 7. Given these results, it can be inferred that in an electron-rich medium, various oxygen radicals interact simultaneously.

### 2.4. Other Oxygen Species

In this study, the thermochemistry and molecular properties of other radical oxygen species were also calculated. Thus, Table 8 summarizes some common oxygen species typically encountered in systems rich in oxygen radicals.

## 3. Discussion

### 3.1. O*_2_* Species

ROS, such as free radicals, are challenging to isolate and characterize experimentally due to their short life and high reactivity [48]. Nonetheless, the computational calculations conducted in this study allow the prediction of thermodynamic properties like Gibbs free energy (G^°^) and total energy (E^°^). These properties are decisive for understanding the reactions and behavior of ROS in biological and chemical systems. Molecular oxygen (O_2_) is a stable component of air under normal conditions, constituting approximately 20% of its composition [49]. The high-energy electrons in O_2_ contribute to their distinctive characteristics. The triplet state of oxygen molecular, O_2_(T), exhibits two unpaired electrons in each of its two antibonding π orbitals (πx* and πy*), both at the same energy level. This configuration imparts O_2_(T) with notable, although non-extreme, reactivity despite being its ground state [50].

The results present in Table 1 indicate that the superoxide anion (^•^O_2_^−^) exhibits the highest energetic stability among the evaluated reactive oxygen species (ROS), indicating that its formation is thermodynamically favorable under photocatalytic conditions. Furthermore, its higher dipole moment suggests a stronger interaction with the solvent, which may impact its reactivity in aqueous environments. Table S1 shows a comparison of the thermochemical values and properties of oxygen molecules calculated using both MP2/TZVP and DFT/B3LYP/TZVP.

Partial reduction in molecular oxygen (O_2_) can lead to the formation of various reactive oxygen species (ROS). Predominant among these are the superoxide radical, ^•^O_2_^−^(D), in a doublet state, and hydrogen peroxide (H_2_O_2_), both of which can be successively formed by the reduction of O_2_(T) or the single-electron filling of the two π* orbitals. Conversely, singlet oxygen, ^1^O_2_(S), is an exceptionally reactive oxygen species generated through a range of chemical and electrochemical reactions. In metal/O_2_ batteries, it is hypothesized that singlet oxygen may arise from superoxide disproportionation [51], interactions with byproducts [52], or in the presence of water or protons which can facilitate its formation [51,53]. Photocatalysis can also lead to the gradual oxidation of water resulting in ^1^O_2_ production [54]. This excited state of oxygen, with its two unpaired electrons in separate orbitals, is highly reactive. Additionally, as a potent oxidant, ^1^O_2_ has a brief half-life, reacting swiftly with proximal compounds, including lipids, proteins, and nucleic acids, before dissipating [55].

In the analysis presented in Table 1, there are visible variations among the molecules in terms of bond distance and vibrational frequencies. However, a notable observation is that they all exhibit the same magnetic moment. This uniformity in magnetic moment suggests a homogeneous distribution of electronic density across these species. Specifically, the electronic spatial extent of the ^•^O_2_^−^(D) radical is observed to be the largest. This is consistent with expectations, as a higher electron density typically leads to an increase in electronic spatial extent. Correspondingly, the O-O bond length in the ^•^O_2_^−^(D) radical is extended, which can be attributed to the electron density effectively pushing the oxygen atoms apart. This increase in bond length is inversely related to the vibrational frequency, leading to its observed decrease.

#### Formation Reactions of O_2_ Species

The energy values from Table 2, indicate that while the formation of ^1^O_2_(S) from O_2_(T) requires an input of 124 kJ mol^−1^ (Reaction (1)), the subsequent formation of ^•^O_2_^−^(D) from ^1^O_2_(S) is spontaneous, releasing almost 400 kJ mol^−1^ (Reaction (2)). For the generation of ^1^O_2_(S) from ^•^O_2_^−^(D) (Reaction (3)), the Haber-Weiss reaction has been proposed [56]. However, this reaction has been reported to have a negligible Gibbs energy change compared to the excitation energy of ^1^O_2_, suggesting that ^1^O_2_ cannot be generated by the Haber-Weiss reaction under these conditions [48]. Alternatively, another pathway (Reaction (4)) that involves an electron (e^−^) can spontaneously generate ^•^O_2_^−^(D) with −281 kJ mol^−1^. It is important to note that the generation of these species involves both an electron (e^−^) and a hole (h^+^), typically provided by a semiconductor material with photocatalytic activity. These semiconductors play a critical role in facilitating these chemical reactions.

As can be seen in Figure 1, the formation of singlet oxygen (^1^O_2_) from triplet oxygen (O_2_) is a complex process, involving energy transfer from excited photosensitizers to ground-state oxygen molecules. In this process, the photosensitizer absorbs light, becoming excited to a higher energy state, and subsequently transfers energy to a ground-state oxygen molecule, elevating it to a singlet excited state [57,58]. This excited singlet oxygen can undergo intersystem crossover to form a more reactive triplet state, which participates in reactions to generate ROS [59]. Additionally, ^1^O_2_ can be generated through the oxidation of superoxide (^•^O_2_^−^), where the superoxide ion is oxidized, forming ^1^O_2_ and a hydroxyl radical (^•^OH) [60].

In photocatalysis, the superoxide radical (^•^O_2_^−^) is produced by transferring electrons from the photocatalyst to the oxygen molecule adsorbed on the photocatalyst surface, a process known as photoinduced reduction of oxygen [61,62,63]. This superoxide radical can also be generated by the Fenton reaction, involving the reduction of hydrogen peroxide (H_2_O_2_) by iron ions (Fe^2+^) under light [64,65].

Notably, the ^•^O_2_^−^(D) species exhibits high oxidative activity and participates in multiple reactions. Multiple experiments show that ^•^O_2_^−^ can dominate oxidative pathways and directly drive contaminant degradation under UV/visible irradiation and co-oxidant assistance. Under visible light, MoSe_2_/PMS systems identified ^•^O_2_^−^ as the primary ROS by scavenger tests and EPR, enabling efficient removal of pharmaceuticals/personal-care products; electron-rich/poor dual sites further promote PMS→^•^O_2_^−^ conversion [66,67]. In UV/K_2_S_2_O_8_ systems, TiO_2_ (P25) boosts O_2_ → ^•^O_2_^−^, overcoming O_2_ inhibition and accelerating carbon tetrachloride degradation (without indiscriminate persulfate activation) [68]. S-doped BiOCl with oxygen vacancies strengthens the built-in electric field, amplifying ^•^O_2_^−^ generation and yielding an 8.8× higher ciprofloxacin degradation rate under visible light [69]. Directional ROS regulation on TiO_2_ (e.g., EDTA-2Na) increases azo-dye degradation consistent with larger ^•^O_2_^−^ contribution [70]. In aerobic photocatalysis, ^•^O_2_^−^ (with ^1^O_2_ and h^+^) is verified by EPR/quenching as a primary oxidant for p-nitrophenol on CQDs@Ag_3_PO_4_ [71]. On nano-TiO_2_, the ^•^O_2_^−^ photogeneration rate correlates with oxygen-vacancy density (chemiluminescence). In Bi@Bi_2_MoO_6_, metallic Bi and oxygen vacancies enhance O_2_^−^-mediated hydroxylated dichlorination and mineralization of sodium pentachlorophenate [72]. Self-sensitized visible-light degradation of oxytetracycline is strongly inhibited by p-benzoquinone, implicating ^•^O_2_^−^ in both direct photolysis and TiO_2_-assisted routes [73]; and in Ag/TiO_2_ heterojunctions, improved carrier separation shifts the dominant oxidant from ^•^OH (pristine TiO_2_) to ^•^O_2_^−^, enhancing dye oxidation.

### 3.2. OH Species

The hydroxyl radical (^•^OH) is a highly reactive and strong oxidizing species, predominantly generated through the oxidation of water (H_2_O) in the presence of light and a photocatalyst like TiO_2_. In semiconductor photocatalysts, absorption of photons with hν ≥ E_g_ promotes electrons from the valence band (VB) to the conduction band (CB), generating electron–hole (e^−^/h^+^) pairs that relax to the band edges within picoseconds. Band bending at the semiconductor–electrolyte interface and surface trap states assist their spatial/energetic separation, while bulk or surface recombination competes with interfacial redox. CB electrons can reduce dissolved O_2_ to O_2_^•−^ (and, downstream, form H_2_O_2_), whereas VB holes oxidize adsorbed H_2_O/OH^−^ to produce ^•^OH (and H^+^ when water is the substrate) [74].

The hydroxyl radical (^•^OH) is extremely reactive and short-lived, making its direct detection difficult. On the other hand, ^•^OH plays a crucial role in photocatalytic systems, actively participating in several oxidation pathways that could contribute to both pollutant removal and, under suboptimal conditions, to the formation of transformation byproducts [75]. According to the literature, in aqueous photocatalysis, ^•^OH-based AOPs—including TiO_2_/UV, UV/H_2_O_2_, and (photo-)Fenton—achieve high removal and mineralization efficiencies for recalcitrant organics [76]. Representative UV/visible tests with TiO_2_ report ≈95% degradation of trichloroethylene (TCE) and tetrachloroethylene (PCE) within ≤150 min using a commercial reactor (Trojan UVMax; emission maxima at 254/436/546 nm), confirming the operational role of ^•^OH in removing chlorinated volatile organic compounds (VOCs); in O_3_–TiO_2_/UV configurations (365 nm LED), optimization of ozone flow, dose, and irradiance enables near-complete removal of dichloroethylene/trichloroethylene/tetrachloroethylene (DCE/TCE/PCE) while suppressing by-product formation through sufficient ^•^OH generation. Nevertheless, the same extreme reactivity of ^•^OH can promote undesired chlorination pathways in chloride-rich matrices (e.g., addition of ^•^OH to PCE with downstream formation of chlorinated alkanes/phosgene), underscoring the need to control operating conditions and co-oxidants to maximize removal and minimize (re)generation of contaminants [77]. These observations agree with the mechanistic picture of ROS generation/detection reported by others authors and with recent advances that structurally regulate photocatalysts to optimize ^•^OH-formation pathways and accelerate rate-limiting steps [21,59], reinforcing the centrality of ^•^OH in removal while explaining its possible contribution to intermediate formation when mineralization is not achieved.

The results shown in Table 4 suggest that under similar conditions, the OH^−^ ion is more stable than the ^•^OH, with the formation of ^•^OH from OH^−^ requiring almost 450 kJ mol^−1^. This view is supported by ultrafast RIXS/XFEL experiments in liquid water, which detect ^•^OH(aq) only under intense excitation and on fs–ps lifetimes [78]. Under ordinary aqueous conditions, OH^−^ is the thermodynamically and kinetically dominant species, and ^•^OH emerges only under very strong oxidizing/excitation conditions, in line with our large positive free energy for OH^−^ → ^•^OH.

While the vibrational frequency values and the O-H bond distance (d_O-H_) are indistinguishable between the two species, there are significant differences in their magnetic moment and electronic spatial extent. These differences highlight the distinct nature of the OH^−^ ion and ^•^OH, underlining the variability in their stability and reactivity. Table S2 shows a comparison of the thermochemical values and properties of hydroxyl species calculated using both MP2/TZVP and DFT/B3LYP/TZVP.

#### Formation Reactions of OH Species

Particularly, the generation of ^•^OH from H_2_O_2_ requires a relatively moderate energy input of 193 kJ mol^−1^. This energy requirement is significantly lower than the energy needed to form ^•^OH from the hydroxide ion (OH^−^), which is about 444 kJ mol^−1^. However, when H_2_O_2_ undergoes reduction with an electron, the energy requirement is further reduced to 39 kJ mol^−1^, making it a more energetically favorable reaction for ^•^OH formation [75]. Apart from these pathways, there exist other routes for generating ^•^OH that require even less energy. However, these alternative pathways often result in the formation of the OH^−^ ion as a byproduct [42]. This indicates a trade-off between the energy efficiency of the reaction and the purity of the ^•^OH produced.

### 3.3. H_2_O_2_ Species

Some reports on the characterization of H_2_O_2_ species revealed that besides the neutral form (H_2_O_2_), it has anionic (H_2_O_2_^−^) and cationic (H_2_O_2_^+^) counterparts. The H_2_O_2_^+^ ion can be generated through the removal of an electron from a H_2_O_2_ molecule, effectively creating a positively charged species. Conversely, the H_2_O_2_^−^ ion forms when a H_2_O_2_ molecule acquires an additional electron, resulting in a negatively charged species. These ionization processes can occur in high-energy environments, such as in photoelectron spectroscopy experiments, or in chemical reactions involving reactive species [79,80]. These forms, particularly H_2_O_2_^−^ and H_2_O_2_^+^, although less explored but potentially play a significant role in photocatalytic reactions.

The results in Table 5 demonstrate a correlation between electronic charge and bond distance, as well as vibrational frequency. Higher electronic charge results in longer bond distances and higher vibrational frequency values (ν_O-H_), alongside lower vibrational frequency values (ν_O-O_). Changes in the H‒O bond distance (d_O-H_) across these molecules are not significant. The electronic charge in the H_2_O_2_^−^ species causes a notable separation between the oxygen atoms, aligning with findings from another research [81]. Table S3 shows a comparison of the thermochemical values and properties of H_2_O_2_ species calculated using both MP2/TZVP and DFT/B3LYP/TZVP.

As shown in Figure 3, the cationic (H_2_O_2_^+^) and anionic (H_2_O_2_^−^) forms of hydrogen peroxide exhibit distinct molecular structures compared to the neutral H_2_O_2_ molecule. These structural variations arise mainly from the addition or removal of an electron in the H_2_O_2_ molecule. Thus, the alteration in the electrical charge significantly impacts the distribution of electronic density within the molecule, causing consequent changes in its molecular structure [81].

The comparative analysis of the H_2_O_2_ species in Figure 3 allows us to clarify the dynamics of photocatalysis, particularly in the context of their roles in environmental remediation processes. The distinct properties of OH^−^ and ^•^OH, such as stability, reactivity, and electronic characteristics, are key factors that influence their effectiveness in various chemical reactions, including those involved in the degradation of pollutants.

#### 3.3.1. Formation Reactions of H_2_O_2_ Species

As can be seen in Table 6, the most spontaneous and energetically favorable reactions (Reactions (16) and (17)) involve electrons and holes, whereas reactions involving water and an oxygen molecule (Reaction (11)) or HO_2_^−^ (Reaction (8)) require energy input, consistent with literature findings [82,83]. Notably, the spontaneous formation of H_2_O_2_ from ^•^O_2_^−^ in an acidic medium (Reaction (16)) aligns with previous studies. The formation of H_2_O_2_ from two ^•^OH (Reaction (15)) is more spontaneous than from an ^•^OH and an OH^−^ ion (Reaction (14)), underscoring the higher reactivity of the ^•^OH. The H_2_O_2_ molecule could be formed from ^•^O_2_^−^ radical [84] but if there are not electrons and holes (Reactions (7), (10) and (12)), the formation will not be spontaneous.

Within the present theoretical scope, these energy profiles map directly onto experiment-relevant levers for targeted H_2_O_2_ synthesis: (i) employ mildly acidic electrolytes to supply H^+^ and steer HO_2_^•^/O_2_^•−^ speciation toward the exergonic Reaction (16); (ii) maintain high dissolved O_2_ and electron-rich operation (illumination/photo-bias or electron-donating environments) to sustain the net two-electron route to H_2_O_2_ rather than competing pathways; (iii) suppress decomposition by avoiding or passivating Fenton-active sites when ^•^OH is not the target and by minimizing O–O–cleaving surfaces; and (iv) tune interfacial adsorption/charge to stabilize peroxo-like intermediates without over-binding that would promote O–O cleavage. For benchmarking, H_2_O_2_ can be quantified with standard assays (including catalase controls) and radical signatures monitored by EPR spin-trapping, while reporting pH, O_2_ availability, and light/electron flux to enable comparison with the thermodynamic predictions. These recommendations are consistent with the pH-driven redox/speciation and interfacial adsorption effects documented experimentally for photocatalysis [20].

#### 3.3.2. Dissociation Reactions of H_2_O_2_ Species

As can be seen in Table 7, dissociation of H_2_O_2_ with h^+^ is spontaneous, often forming water, oxygen, or other species. Reaction (19) indicates that the presence of e^−^ renders H_2_O_2_ dissociation non-spontaneous. The spontaneity of certain reactions underscores the necessity of radicals in maintaining ROS in the environment, as exemplified by the interaction of H_2_O_2_ and OH^−^ ion yielding water. Furthermore, Reaction (21) suggests that H_2_O and O_2_ molecules are more stable than H_2_O_2_, as H_2_O_2_ spontaneously dissociates into water and molecular oxygen. Conversely, H_2_O_2_ is more stable than both the ^•^OH and OH^−^ ion.

The environment of photochemical reactions that involve reactive oxygen species (ROS) is inherently complex, influenced by various factors such as temperature, impurities, and the composition of the medium. This complexity presents challenges in isolating and analyzing the effects of each factor. For instance, the extremely short lifetimes of these species, the potential for parallel and consecutive reactions, and the influence of photocatalytically active semiconductor materials and radiation sources all contribute to the formation of various reactive species by providing available electrons and holes. These factors can significantly alter the original species, leading to the generation of new ROS [85], some of which are presented in Table 8. Table S4 shows a comparison of the thermochemical values and properties of several radical oxygen species calculated using both MP2/TZVP and DFT/B3LYP/TZVP.

When considering the potential reactions among these species, numerous routes involving ROS in photocatalysis have been hypothesized. However, it is crucial to recognize that the actual reactions occurring near the surface are limited by adsorption phenomena and specific electrical charges. Photocatalysis typically involves simultaneous oxidation and reduction processes, with ROS being produced sequentially from both O_2_ and H_2_O. For instance, the stepwise oxidation of H_2_O can sequentially generate ROS such as hydroxyl radicals (^•^OH), hydrogen peroxide (H_2_O_2_), superoxide anion (^•^O_2_^−^), and singlet oxygen (^1^O_2_). Conversely, the stepwise reduction of O_2_ can lead to the formation of species like superoxide anion (^•^O_2_^−^), hydrogen peroxide (H_2_O_2_), and hydroxyl radicals (^•^OH) [86]. This intricate interplay of reactions dictates the overall effectiveness of photocatalytic processes, and therefore a comprehensive understanding of these pathways is essential to optimize photocatalytic systems for environmental and energy applications.

**Figure 7:**
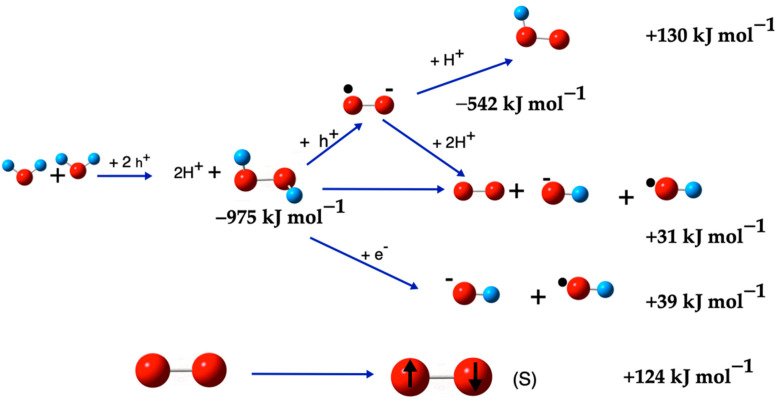
ROS generation from H_2_O or O_2_ molecules, the energy of each step is indicated, but not the final energy of the full reaction, the total energy is shown in Table 9. The red balls are oxygen atoms and small blue balls are hydrogen atoms, the small black ball mark OH as a radical, and the negative sign as super-index indicates that the species has negative charge.

Finally, throughout this work, reactions of ROS formation from various species have been described, as well as dissociation reactions of ROS to form other molecules. Both groups of reactions involve many species. It should not be forgotten that in a photocatalytic system, there are many highly reactive species, such as ROS and, with favorable conditions for high chemical activity, direct and reverse reactions should occur simultaneous, parallel and consecutively, forming multiple species, among others, short-life intermediates; in this scenario, it is very difficult to draw unequivocal conclusions. We summarize the results in Table 9 and Figure 7, highlighting that those reactions are more energetically favored and, therefore, the most probable ones to form the interesting ROS.

Table 9 and Figure 7 show that H_2_O_2_ appears in the early stages of the ROS cascade. Experimentally, in aqueous photocatalysis, H_2_O_2_ can arise through two supported routes: (i) two-hole oxidation of interfacial H_2_O/OH^−^ to surface ^•^OH followed by 2^•^OH → H_2_O_2_ (the net stoichiometry corresponds to Reaction (17)), and (ii) two-electron reduction in dissolved O_2_ by conduction-band electrons via ^•^O_2_^−^/HO_2_^•^ → H_2_O_2_. Under deoxygenated conditions, route (i) dominates, whereas in aerated solutions route (ii) is often predominant. Apart from ^1^O_2_ (formed by energy transfer), H_2_O_2_ is a key intermediate in the formation of other ROS, but its immediate origin (water vs. oxygen) depends on O_2_ levels, pH, and the catalyst surface (phase, trapping/adsorption) [20,87].

## 4. Computational Methods

The study of the formation reactions of various reactive oxygen species (ROS) was conducted using Moller-Pleset 2 (MP2) [88,89,90] as implemented in Gaussian version 16 software package [91] (Gaussian, Inc., Wallingford, CT, USA) for determine the thermochemical properties of the ROS. This method describes accurately electronic interactions, particularly important in the chemistry of reactive species like ROS [92,93,94,95].

In this study, second-order Møller–Plesset perturbation theory (MP2) was prioritized for mapping the thermodynamics of ROS, as it provides an explicit description of dynamic electron correlation and has demonstrated accuracy and computational efficiency [38]. MP2 serves as a post-Hartree–Fock alternative when approximate DFT functionals can incur self-interaction and delocalization errors in charge-separated radical states [96]. In this context, for the small ROS described in this study, MP2 offers a favorable balance between accuracy and cost and produces thermodynamic trends consistent with a lower functional dependence on aqueous solvation [97]. The Supplementary Materials (Tables S1–S4) comparatively document the thermochemistry and molecular properties of MP2/TZVP and DFT/B3LYP/TZVP.

The molecular properties of the species studied were computed using the Ahlrichs et al. basis set, specifically the triple-zeta valence with polarization (TZVP) basis set [98,99]. This set was chosen for its ability to accurately represent the electronic structure of the species. The TZVP basis set is optimized to balance the precision of valence orbital descriptions with computational efficiency [100]. This approach ensures a reliable depiction of key elements, allowing for accurate predictions regarding the stability and reactivity of the species. The choice of the TZVP basis set underpins the reliability of this study, providing precise insights into the molecular properties of reactive oxygen species (ROS).

In this study, the root mean square convergence criterion for the density matrix in the self-consistent field (SCF) iteration was set to 10^−14^ a.u., aiming for an energy convergence threshold of at least 10^−15^ a.u. (GAUSSIAN keyword: SCF = tight). This default convergence criteria recommended by Gaussian 16 were applied to ensure the reliability and precision in the calculations. These criteria ensure that the resulting structures represent appropriate energy minima, which is an important aspect for studies aiming to understand the stability and reaction pathways of ROS. It is worth mentioning that the total energy, defined here as the sum of all electronic contributions plus zero-point energy corrections, is an important metric to evaluate the thermodynamic stability and chemical reactivity of ROS. The precision in the determination of the total energy allows direct comparisons between different chemical species and the evaluation of possible reaction pathways and mechanisms.

After the geometric optimization of the molecules, which aims to locate the structures at their most stable energy minima, it proceeded with the calculation of the vibrational frequencies. Vibrational frequency calculations are critical for confirming that optimized geometries represent true energy minima, as indicated by the absence of imaginary frequencies. These calculations provide insights into molecular stability and potential reaction mechanisms, enhancing our understanding of ROS dynamics. Secondly, it provides valuable insights into molecular dynamics, allowing a better understanding of how ROS interact and react in various contexts. The vibrational frequencies were calculated by applying the principle of harmonic oscillation, which assumes that molecular vibrations near equilibrium can be modeled as harmonic oscillators. Gaussian 16 uses advanced algorithms to determine force constants from which frequencies are calculated.

The analysis of vibrational frequencies offers detailed information on the rigidity of molecular bonds and the stability of structures. Higher frequencies indicate stronger bonds and more rigid structures, while lower frequencies may indicate weaker bonds or more flexible molecular groups. This analysis is complemented by the calculation of the zero-point energy (ZPE) and thermal corrections to thermodynamic properties such as enthalpy and Gibbs free energy, which are critical to understanding the thermodynamics of the reactions in which ROS participate. By integrating the Polarizable Continuum Model (PCM), it was possible to assess the impact of the solvent environment on vibrational frequencies, which is crucial for accurate simulations of molecular dynamics in aqueous solutions.

The PCM simulates the solvent as a continuous polarizable medium surrounding the solute, automatically generating a virtual cavity based on the molecular geometry of the study molecule, ensuring an accurate representation of the polarizing effect of the solvent. The selection of water as a solvent in all simulations was based on its relevance in biological and photocatalytic processes [20]. Gaussian 16 allows water to be specified as a solvent by adjusting its dielectric constant (ε = 78.4), together with the surface tension and other relevant solvent parameters, to values characteristic of water at room temperature. These adjustments are essential to align the simulations with real experimental conditions, ensuring that the computational findings are applicable and relevant to the analysis of ROS interactions and reactions under typical experimental conditions [101,102].

Finally, visualization of all molecular structures and properties was enabled by the GaussView version 6 software package (Semichem Inc., Shawnee Mission, KS, USA) [103].

## 5. Conclusions

In this computational study, the different molecular oxygen (O_2_) species were examined, including O_2_(T) in its triplet state, ^1^O_2_(S) in its singlet state, and ^•^O_2_^−^(D) in its negatively charged doublet state. The results indicate that these species possess similar molecular properties, such as bond lengths and dipole moments, suggesting a uniform distribution of electron density across these molecules. However, notable distinctions in bond distances and vibrational frequencies were observed, which may significantly impact their reactivity and chemical behavior. A key finding is that the ^•^O_2_^−^(D) species exhibits the largest electronic spatial extent, aligning with its increased electron density due to its negative charge. This attribute potentially renders it highly reactive in chemical reactions.

In terms of the interconversion of these molecular oxygen species, this study reveals that the transformation from O_2_(T) to O_2_(S) requires energy, indicating its non-spontaneity under the examined conditions. Conversely, the conversion of O_2_(S) to ^•^O_2_^−^(D) is a spontaneous reaction, releasing substantial energy, approximately 400 kJ mol^−1^.

This research also examined hydrogen peroxide (H_2_O_2_) in its neutral, anionic, and cationic forms, revealing marked differences in their thermodynamic feasibility that depend on the surrounding environment. Computed maps indicate that the anionic pathway is thermodynamically accessible under electron-rich conditions and is favored at alkaline pH (H_2_O_2_/HOO^−^ speciation), whereas the cationic form is disfavored, consistent with its expected rarity. Within the theoretical scope adopted here, the exergonic H_2_O_2_-formation routes are consistent with pH-driven redox/speciation and interfacial adsorption/charge effects reported experimentally, thereby delineating clear levers for benchmarking: pH control (mildly acidic to accumulate H_2_O_2_; alkaline to probe HOO^−^), dissolved O_2_ availability, and surface properties (adsorption/charge) that stabilize peroxo-like intermediates [20]. For experimental verification, validation can rely on standard H_2_O_2_ assays (with catalase controls) and EPR spin-trapping for radicals; reporting operating parameters (pH, O_2_, light/electron flux) is recommended to enable direct comparison with the present thermodynamic predictions [20].

The study further explored a range of radical oxygen species present in ROS-rich systems, defined by elevated interfacial production and higher steady-state levels of ROS (^•^OH, ^•^O_2_^−^/HO_2_^•^, H_2_O_2_, ^1^O_2_) relative to dark or no-catalyst controls. A diversity of molecular properties was observed among these species, including variations in bond lengths, vibrational frequencies, and dipole moments. The presence of H^+^ ions, indicative of medium acidity, appears to be a critical factor influencing many reactions involving these species. Additionally, we investigated potential reactions among these radical oxygen species, particularly focusing on the highly reactive ^•^O_2_^−^(D) species. A variety of reactions, leading to both spontaneous and non-spontaneous products, was observed, emphasizing the intricate network of chemical interactions in environments rich in oxygen radicals.

Finally, the results of this study provide an intrinsic aqueous-phase thermodynamic map (ΔE/ΔG) and molecular descriptors for key ROS (^•^OH, ^•^O_2_^−^/HO_2_^•^, H_2_O_2_, ^1^O_2_), identifying exergonic vs. endergonic steps and the pH-conditioned speciation that govern their interconversion. Although derived for air-saturated water at 298 K without explicit surfaces, this baseline constrains which pathways are thermodynamically feasible in chemically and biologically complex milieus, where microenvironmental pH/[H^+^], O_2_ availability, interfacial adsorption, and metal centers (e.g., Fe/Cu) modulate kinetics and selectivity. The framework therefore offers testable predictions and design guidance for photocatalytic and AOP settings (e.g., UVA-driven semiconductor interfaces or UVC/UV–H_2_O_2_ systems) and a quantitative basis for interpreting ROS-mediated processes in cells and environmental matrices.

## Figures and Tables

**Table 1:** Thermochemistry and calculated molecular properties of oxygen molecules. Between parenthesis, the multiplicity of each species is described (T = triplet, S = singlet and D = doublet). The (^•^) symbol indicates that the species is radical and the negative sign as super-index indicates that the species has negative charge.

Species	E^°^ (Hartree)	G^°^ (Hartree)	d_O-O_ (Å)	ν_O-O_ (cm^−1^)	μ (Debye)	**R^2^** **(Å^2^)**
O_2_(T)	−150.04	−150.06	1.23	1423	0	12.43
^1^O_2_(S)	−149.99	−150.01	1.26	1216	0	12.75
^•^O_2_^−^(D)	−150.15	−150.17	1.37	1064	0	16.02

**Table 2:** Possible reactions between the three oxygen species. The energy difference between species in reactions is obtained from the difference between the sum of the energy of the reaction products and the sum of the energy of the reactants.

Reaction		ΔE (kJ mol^−1^) *	ΔG (kJ mol^−1^)
O_2_(T) → ^1^O_2_(S)	(1)	124	126
^1^O_2_(S) → ^•^O_2_^−^(D) + e^−^	(2)	−405	−407
^•^O_2_^−^(D) + h^+^ → ^1^O_2_(S)	(3)	405	407
O_2_(T) + e^−^ → ^•^O_2_^−^(D)	(4)	−281	−281

* The energy difference between species in reactions is obtained from the difference between the sum of the total energy or free energy of the reaction products and the sum of the total energy or free energy of the reactants.

**Table 3:** Possible reactions between radical oxygen species with the ^•^O_2_^−^(D) species as reactive.

Reaction		ΔE (kJ mol^−1^)	ΔG (kJ mol^−1^)
^•^O_2_^−^ + ^•^OH → O_2_(S) + OH^−^	(5)	−38	−34
^•^O_2_^−^ + H^+^ → ^•^O_2_H	(6)	130	152

**Table 4:** Thermochemistry and calculated molecular properties of hydroxyl species.

Species	E^°^ (Hartree)	ΔE^°^ (kJ mol^−1^)	G^°^ (Hartree)	ΔG^°^ (kJ mol^−1^)	d_O-H_ (Å)	ν_O-H_ (cm^−1^)	μ (Debye)	R^2^ (Å^2^)
OH^−^(S)	−75.75	−444	−75.77	−442	0.96	3794	6.23	
^•^OH(D)	−75.58		−75.60		0.97	3826	2.11	

**Table 5:** Thermochemistry and molecular properties of H_2_O_2_ species. ΔG^°^ is calculated from the difference between the negative or positive species and the neutral one. The units of bond distances are in Å and the vibrational frequency is in cm^−1^.

Species	E^°^ (Hartree)	G^°^ (Hartree)	ΔG^°^ (kJ mol^−1^)	d_O-O_ (Å)	d_O-H_ (Å)	ν_O-H_ (cm^−1^)	ν_O-O_ (cm^−1^)	μ (Debye)	R^2^ (Å^2^)
H_2_O_2_	−151.23	−151.26		1.46	0.97	3799 3803	931	2.48	47.1
H_2_O_2_^−^	−151.34	−151.37	−295	2.19	0.97	3854 3857	367	1.55	32.4
H_2_O_2_^+^	−151.94	−151.96	773	1.31	1.00	3541	896	3.49	15.8

**Table 6:** Possible chemical reactions for the generation of H_2_O_2_.

Reaction		ΔE (kJ mol^−1^)	ΔG (kJ mol^−1^)
^•^O_2_^−^ + 2H^+^ → H_2_O_2_ + h^+^	(7)	542	590
HO_2_^−^ + H_2_O → H_2_O_2_ + OH^−^	(8)	492	494
^•^O_2_H + H^+^ → H_2_O_2_ + h^+^	(9)	412	438
^•^O_2_^−^ + ^•^O_2_H + H^+^ → O_2_(S) + H_2_O_2_	(10)	43	73
½ O_2_ + H_2_O → H_2_O_2_	(11)	127	142
^•^O_2_^−^ + ^•^O_2_H + H_2_O → H_2_O_2_ + O_2_ + OH^−^	(12)	−45	−40
^•^OH + O_2_ + OH^−^ → H_2_O_2_ + O_2_^−^	(13)	−31	−2
^•^OH + OH^−^ → H_2_O_2_ + e^−^	(14)	−39	−16
^•^OH + ^•^OH → H_2_O_2_	(15)	−193	−163
^•^O_2_^−^ + 2H^+^ + e^−^ → H_2_O_2_	(16)	−232	−182
2H_2_O + 2h^+^ → H_2_O_2_ + 2H^+^	(17)	−975	−990

**Table 7:** Possible chemical reactions for the dissociation of H_2_O_2_.

Reaction		ΔE (kJ mol^−1^)	ΔG (kJ mol^−1^)
H_2_O_2_ → ^•^OH + ^•^OH	(18)	193	163
H_2_O_2_ + e^−^ → ^•^OH + OH^−^	(19)	39	16
H_2_O_2_ + ^•^O_2_^−^ → ^•^OH + O_2_ + OH^−^	(20)	31	2
H_2_O_2_ → ½ O_2_ + H_2_O	(21)	−127	−142
H_2_O_2_ + h^+^ → ^•^O_2_H + H^+^	(22)	−412	−438
H_2_O_2_ + OH^−^ → HO_2_^−^ + H_2_O	(23)	−492	−494
H_2_O_2_ + h^+^ → ^•^O_2_^−^ + 2H^+^	(24)	−542	−590

**Table 8:** Thermochemistry and molecular properties of several radical oxygen species. ΔG^°^ is calculated from the difference between the negative or positive species and the neutral one. The units of bond distances are in Å and the vibrational frequency is in cm^−1^.

Species	E^°^ (Hartree)	G^°^ (Hartree)	d_O-O_ (Å)	d_O-H_ (Å)	ν_O-H_ (cm^−1^)	ν_O-O_ (cm^−1^)	μ (Debye)	R^2^ (Å^2^)
H^+^	−0.50	−0.51					0	3.1
O^−^(S)	−75.08	−75.09						4.8
H_2_O(S)	−76.26	−76.28		0.96	3844 3952	1626	2.50	29.3
H_2_O^+^(D)	−75.93	−75.95		1.00	3493 3543	1483	6.28	25.6
^•^O_2_H(D)	−150.60	−150.62	1.33	0.98	3679	1226 1441	2.40	43.9
O_2_H^−^(S)	−150.74	−150.76	1.51	0.96	3839	1177 891	9.28	50.6

**Table 9:** The most possible chemical reactions for the ROS formation from molecules.

Target ROS	Reaction		ΔE (kJ mol^−1^)	ΔG (kJ mol^−1^)
^•^O_2_H	2H_2_O + 2h^+^ → H_2_O_2_ + 2H^+^ H_2_O_2_ + h^+^ → ^•^O_2_^−^ + 2H^+^ ^•^O_2_^−^ + H^+^ → ^•^O_2_H	(17) (24) (6)	−975 −542 +130 ΔE = −1387	−990 −590 +152 ΔG = −1428
H_2_O_2_	2H_2_O + 2h^+^ → H_2_O_2_ + 2H^+^	(17)	−975	−990
^•^O_2_^−^	2H_2_O + 2h^+^ → H_2_O_2_ + 2H^+^ H_2_O_2_ + h^+^ → ^•^O_2_^−^ + 2H^+^	(17) (24)	−975 −542 ΔE = −1517	−990 −590 ΔG = −1580
^•^OH	2H_2_O + 2h^+^ → H_2_O_2_ + 2H^+^ H_2_O_2_ + h^+^ → ^•^O_2_^−^ + 2H^+^ H_2_O_2_ + ^•^O_2_^−^ → ^•^OH + O_2_ + OH^−^	(17) (24) (20)	−975 −542 +31 ΔE = −1486	−990 −590 +2 ΔG = −1578
^•^OH	2H_2_O + 2h^+^ → H_2_O_2_ + 2H^+^ H_2_O_2_ + e^−^ → ^•^OH + OH^−^	(17) (19)	−975 +39 ΔE = −936	−990 +16 ΔG = −974
^1^O_2_	O_2_ → ^1^O_2_	(1)	+124	+126

## Data Availability

Data are available within the article.

## Supplementary Materials

The following supporting information can be downloaded at: https://www.mdpi.com/article/10.3390/ijms26188989/s1.

## Abbreviations

The following abbreviations are used in this manuscript:

ROS	Reactive Oxygen Species
**^•^**O_2_^−^	Superoxide anion radical
H_2_O_2_	Hydrogen peroxide
^1^O_2_	Singlet oxygen
**^•^**OH	Hydroxyl radical
H_2_O_2_^+^	Protonated hydrogen peroxide
H_2_O_2_^−^	Deprotonated hydrogen peroxide
OH^−^	Hydroxide ion
H^+^	Hydrogen ion or proton
H_2_O	Water
HO_2_^−^	Perhydroxide ion
**^•^**O_2_H	Hydroperoxyl radical
e^−^	Electron
h^+^	Hole
T	Triplet state
S	Singlet state
D	Doublet state
G°, E°	Standard Gibbs free energy and total energy
ΔG	Change in Gibbs Free Energy
ΔE	Change in Energy
ΔG^0^	Standard change in Gibbs Free Energy
dO-O, dO-H	Bond distances between O atoms and between O and H atoms
νO-O, νO-H	Vibrational frequencies of O-O and O-H bonds
μ	Dipole moment
R^2^	Electronic spatial extent
ZPE	Zero-Point Energy
B3LYP	Becke, 3-parameter, Lee-Yang-Parr
SCF	Self-Consistent Field
PCM	Polarizable Continuum Model
MP2	Moller-Pleset 2
