# Supplementary material for: Ab Initio Study of Formation Mechanisms and Thermochemical Properties of Reactive Oxygen Species (ROS) in Photocatalytic Processes
Source: Int J Mol Sci. 2025 Sep 15;26(18):8989. doi: 10.3390/ijms26188989 (PMC12469441; doi:10.3390/ijms26188989)
Supplement: Supplementary file 1 [file ijms-26-08989-s001.zip › ijms-3826869-supplementary.pdf]

International Journal of *Molecular Sciences*

MDPI

Supplementary Material

Ab Initio Study of Formation Mechanisms and Thermochemical Properties of Reactive Oxygen Species (ROS) in Photocatalytic Processes

Silvia González and Ximena Jaramillo-Fierro *

Departamento de Química, Facultad de Ciencias Exactas y Naturales, Universidad Técnica Particular de Loja, San Cayetano Alto, Loja 1101608, Ecuador; sgonzalez@utpl.edu.ec
* Correspondence: xvjaramillo@utpl.edu.ec; Tel.: +593-7-3701444

Table S1. Thermochemistry and properties of oxygen molecules calculated using MP2/TZVP and DFT/B3LYP/TZVP.

Method	Species	E^0 (hartree)	G^0 (hartree)	$d_{O\text{-}O}$ (Å)	$\nu_{O\text{-}O}$ (cm^{-1})	μ (Debye)	R^2 (Å^2)
MP2/ TZVP	O_2(T)	-150.04	-150.06	1.23	1423	0	12.43
	$^1O_2$(S)	-149.99	-150.01	1.26	1216	0	12.75
	$^{\bullet}O_2^-$(D)	-150.15	-150.17	1.37	1064	0	16.02
DFT/ B3LYP/ TZVP	O_2(T)	-150.38	-150.40	1.21	1620	0	13.40
	$^1O_2$(S)	-150.32	-150.34	1.21	1609	0	12.30
	$^{\bullet}O_2^-$(D)	-150.51	-150.53	1.35	1175	0	15.90

Table S2. Thermochemistry and calculated molecular properties of hydroxyl species calculated using MP2/TZVP and DFT/B3LYP/TZVP.

Method	Species	E^0 (hartree)	G^0 (hartree)	$d_{O\text{-}H}$ (Å)	$\nu_{O\text{-}H}$ (cm^{-1})	μ (Debye)	R^2 (Å^2)
MP2/ TZVP	OH^-(S)	−75.75	−75.77	0.96	3794	6.23	14.28
	$^{\bullet}OH$(D)	−75.58	−75.60	0.97	3826	2.11	12.00
DFT/ B3LYP/ TZVP	OH^-(S)	-75.94	-75.95	0.97	3748	6.15	14.20
	$^{\bullet}OH$(D)	-75.76	-75.78	0.99	3678	2.03	11.80

 https://doi.org/10.3390/ijms26188989

Table S3. Thermochemistry and molecular properties of H_2O_2 species calculated using MP2/TZVP and DFT/B3LYP/TZVP.

Method	Species	E^0 (hartree)	G^0 (hartree)	$d_{O\text{-}O}$ (Å)	$d_{O\text{-}H}$ (Å)	$\nu_{O\text{-}H}$ (cm^{-1})	$\nu_{O\text{-}O}$ (cm^{-1})	μ (Debye)	R^2 ($Å^2$)
MP2/ TZVP	H_2O_2	−151.23	−151.26	1.46	0.97	3799<br>3803	931	2.48	47.1
	$H_2O_2^-$	−151.34	−151.37	2.19	0.97	3854<br>3857	367	1.55	32.4
	$H_2O_2^+$	−151.94	−151.96	1.31	1.00	3541	896	3.49	15.8
DFT/ B3LYP/ TZVP	H_2O_2	-151.59	-151.61	1.45	0.97	3743<br>3749	948	2.43	47.3
	$H_2O_2^-$	-151.73	-151.76	2.31	0.96	3807<br>3812	302	1.16	35.0
	$H_2O_2^+$	-151.31	-151.33	1.31	1.00	3400<br>3463	1239	3.49	15.6

Table S4. Thermochemistry and molecular properties of several radical oxygen species calculated using MP2/TZVP and DFT/B3LYP/TZVP.

	Species	E^0 (hartree)	G^0 (hartree)	$d_{O\text{–}O}$ (Å)	$d_{O\text{–}H}$ (Å)	$\nu_{O\text{–}H}$ (cm^{-1})	$\nu_{O\text{–}O}$ (cm^{-1})	μ (Debye)	R^2 ($Å^2$)
MP2/ TZVP	H^+	-0.50	-0.51					0	3.1
	O^-(S)	-75.08	-75.09						4.8
	H_2O(S)	-76.26	-76.28		0.96	3844<br>3952	1626	2.50	29.3
	H_2O^+(D)	-75.93	-75.95		1.00	3493<br>3543	1483	6.28	25.6
	$^{\bullet}O_2H$(D)	-150.60	-150.62	1.33	0.98	3679	1226<br>1441	2.40	43.9
	O_2H^-(S)	-150.74	-150.76	1.51	0.96	3839	1177<br>891	9.28	50.6
DFT/ B3LYP/ TZVP	H^+	-0.50	-0.51					0	3.1
	O^-(S)	-75.27	-75.28						4.8
	H_2O(S)	-76.45	-76.47		0.96	3879<br>3786	1610	2.40	29.3
	H_2O^+(D)	-76.11	-76.13		1.00	3420<br>3384	1463	6.30	25.6
	$^{\bullet}O_2H$(D)	-150.96	-150.98	1.33	0.98	3588	1419<br>1166	2.83	44.6
	O_2H^-(S)	-151.11	-151.13	1.51	0.96	3777	1163<br>825	9.73	51.3
